# Risk of Transmission of Antimicrobial Resistant *Escherichia coli* from Commercial Broiler and Free-Range Retail Chicken in India

**DOI:** 10.3389/fmicb.2017.02120

**Published:** 2017-11-13

**Authors:** Arif Hussain, Sabiha Shaik, Amit Ranjan, Nishant Nandanwar, Sumeet K. Tiwari, Mohammad Majid, Ramani Baddam, Insaf A. Qureshi, Torsten Semmler, Lothar H. Wieler, Mohammad A. Islam, Dipshikha Chakravortty, Niyaz Ahmed

**Affiliations:** ^1^Department of Microbiology and Cell Biology, Indian Institute of Science, Bengaluru, India; ^2^Pathogen Biology Laboratory, Department of Biotechnology and Bioinformatics, University of Hyderabad, Hyderabad, India; ^3^International Centre for Diarrhoeal Disease Research Bangladesh (icddr,b), Dhaka, Bangladesh; ^4^Department of Biotechnology and Bioinformatics, School of Life Sciences, University of Hyderabad, Hyderabad, India; ^5^Robert Koch Institute, Berlin, Germany

**Keywords:** food borne pathogens, poultry, antibiotic resistance, zoonosis, whole genome sequencing

## Abstract

Multidrug-resistant *Escherichia coli* infections are a growing public health concern. This study analyzed the possibility of contamination of commercial poultry meat (broiler and free-range) with pathogenic and or multi-resistant *E. coli* in retail chain poultry meat markets in India. We analyzed 168 *E. coli* isolates from broiler and free-range retail poultry (meat/ceca) sampled over a wide geographical area, for their antimicrobial sensitivity, phylogenetic groupings, virulence determinants, extended-spectrum-β-lactamase (ESBL) genotypes, fingerprinting by Enterobacterial Repetitive Intergenic Consensus (ERIC) PCR and genetic relatedness to human pathogenic *E. coli* using whole genome sequencing (WGS). The prevalence rates of ESBL producing *E. coli* among broiler chicken were: meat 46%; ceca 40%. Whereas, those for free range chicken were: meat 15%; ceca 30%. *E. coli* from broiler and free-range chicken exhibited varied prevalence rates for multi-drug resistance (meat 68%; ceca 64% and meat 8%; ceca 26%, respectively) and extraintestinal pathogenic *E. coli* (ExPEC) contamination (5 and 0%, respectively). WGS analysis confirmed two globally emergent human pathogenic lineages of *E. coli*, namely the ST131 (*H*30-Rx subclone) and ST117 among our poultry *E. coli* isolates. These results suggest that commercial poultry meat is not only an indirect public health risk by being a possible carrier of non-pathogenic multi-drug resistant (MDR)-*E. coli*, but could as well be the carrier of human *E. coli* pathotypes. Further, the free-range chicken appears to carry low risk of contamination with antimicrobial resistant and extraintestinal pathogenic *E. coli* (ExPEC). Overall, these observations reinforce the understanding that poultry meat in the retail chain could possibly be contaminated by MDR and/or pathogenic *E. coli.*

## Introduction

The rapid global rise of *Escherichia coli* infections that are resistant to therapeutically important antimicrobials, including first-line drugs such as cephalosporins and fluoroquinolones, is of serious concern, as it hampers treatment of infections leading to significant morbidity, mortality, medical costs as well as production losses in livestock (de Been et al., 2014). *E. coli* is responsible for infections in humans and animals; these could be nosocomial and/or community-acquired (Jadhav et al., 2011). Being part of the endogenous microbiota, *E. coli* can easily acquire resistance against antimicrobials consumed by humans and animals (van den Bogaard et al., 2001).

Poultry are recognized as important source for dissemination of antimicrobial resistant *E. coli* in the community and environment (van den Bogaard et al., 2001). Pathogenic *E. coli* in poultry are a direct threat to both poultry industry and human health as they may result in hard-to-treat infections (Kaper et al., 2004). Extraintestinal pathogenic *E. coli* (ExPEC), the causative agent of colibacillosis in chickens inflict severe losses due to morbidity and condemnations (Kaper et al., 2004). ExPEC can also cause several extraintestinal diseases in humans, including urinary tract infections, neonatal meningitis, and sepsis (Avasthi et al., 2011; Hussain et al., 2012; Nandanwar et al., 2016; Ranjan et al., 2017). Apart from ExPEC, the poultry gut could also harbor other variants of intestinal pathogenic *E. coli* (Lutful Kabir, 2010). Recent studies in different parts of India have reported antimicrobial residues in food animal products such as milk and chicken meat, indicating that antimicrobial usage is widespread in food animal production (Laxminarayan and Chaudhury, 2016). Such practices lead to high proportion of antibiotic resistant bacteria in their fecal microbiota (Chen and Jiang, 2014). Consequently, meat at slaughtering operations can be extensively contaminated with fecal *E. coli* of poultry origin (Lutful Kabir, 2010).

The ExPEC pathotype that is isolated from poultry with clinical signs of extraintestinal infections is known as avian pathogenic *E. coli* (APEC) (Kaper et al., 2004). Although research has been mainly focused on infections caused by APEC pathotype, little is known about the reservoirs of these bacteria (Bélanger et al., 2011). Also, many human and animal ExPEC isolates share virulence genes and clonal backgrounds and the human health risk posed by such bacteria from poultry is still largely undefined (Dziva et al., 2013). Moreover, the pathotype APEC itself is ill-defined (Collingwood et al., 2014). One study even suggested that an APEC strain showed high genome similarity to human enterotoxigenic *E. coli* (ETEC) (Dziva et al., 2013). Therefore, the question of whether fecal isolates of poultry serve as a source of infection for extraintestinal and intestinal infections in humans remains unanswered. Recent investigations compared *E. coli* from symptomatic poultry and human ExPEC by virulence genotyping, serotyping and *in vitro* assays (Nandanwar et al., 2014; Mitchell et al., 2015). However, the application of high-resolution, whole genome sequencing methods appears to be superior and is likely to provide accurate insights into the phylogenetic backgrounds of poultry *E. coli* and would likely highlight the similarities between isolates of different human pathotypes (de Been et al., 2014).

The presence of antimicrobial resistant bacteria in food has been attributed to the widespread use of antimicrobials in farming practices (Landers et al., 2012; Qumar et al., 2017). Currently, few data are available regarding the contamination of retail foods with *E. coli*, especially those that are multi-resistant and pathogenic (Zhang et al., 2011). Also, little is known about the frequency of antibiotic-resistant microorganisms in poultry that were raised by free-range farming as methods of livestock production differ in antibiotic usage practices (Brower et al., 2017). Therefore, the main aim of this study was to estimate the frequencies of contamination with pathogenic and/or multi resistant *E. coli* among broiler and free-range chicken specimens (ceca and meat) and to characterize the *E. coli* isolates recovered from them in relation to the human *E. coli* pathotypes. Results of this study reinforces the importance of One Health approach in addressing the spread of antimicrobial resistance and emerging infections.

## Materials and Methods

### Specimen Collection

Between February, 2015 to September, 2015, 22 poultry retail outlets were sampled from four cities representatives of four different states of India: Karnataka (*n* = 35), Telangana (*n* = 59), Andhra Pradesh (*n* = 15) and Maharashtra (*n* = 11); this resulted in a total of 120 samples. A total of 75 poultry ceca, entailing broiler and free-range chicken (39 and 36, respectively) together with 45 raw meat samples, representing broiler and free-range chicken (32 and 13, respectively) were obtained from retail poultry outlets. From each shop, multiple samples (different birds) were procured. Samples were transported to laboratory in cooled boxes (4–8°C) and upon arrival were stored at 4°C and processed within 24 h.

Broiler represented commercial broiler chickens that were conventionally raised in farms and fed with commercial feeds; free-range poultry birds were country (native) chickens that were raised in households and small backyard farms that grew by free-ranging.

### Culture Methods

All cecal samples were surface sterilized with 70% ethanol and a portion (∼25 g) was incised and placed in a 1.5 ml micro-centrifuge tube containing Luria Bartani (LB) broth. Similarly, around 25 g of raw meat was excised from each sample and rinsed with 1X PBS and the sample was placed in a Petri dish containing 1 ml LB broth and minced into small pieces. Each cecum and chicken sample was taken out of the LB broth and swabbed on the edge of the plate and then spread with a loop, this was done on two separate agar plates; one on unsupplemented Eosin Methylene Blue (EMB) agar plate, and another on EMB agar supplemented with 10 μg/ml ciprofloxacin together with 4 μg/ml cefotaxime (this was used to increase the chance of ST131 *E. coli* isolation). All plates were incubated at 37°C for 12 h. One putative *E. coli* was selected from each culture plate, which was then confirmed by standard biochemical methods. All *E. coli* isolates were preserved in 20% glycerol-supplemented Luria-Bertani broth at -80°C.

### Antimicrobial Susceptibility, ESBL Confirmation, and ESBL Gene Detection

Antimicrobial susceptibility toward fosfomycin, gentamicin, ciprofloxacin, co-trimoxazole, tetracycline, and chloramphenicol belonging to six different antibiotic classes was assessed by standard disk diffusion method as per CLSI guidelines (Schissler et al., 2009; Hussain et al., 2014). ESBL production was determined using double-disk synergy test following CLSI guidelines (CLSI, 2013). Phenotypically confirmed ESBL-producers were analyzed for the presence of genes encoding CTX-M-15 and that of *bla*_SHV_, and *bla*_TEM_ genes by three gene specific PCRs (Ranjan et al., 2015).

As the EMB agar plates (i.e., with or without antibiotic supplement) were streaked using an identical inoculum, the percentage of resistance/susceptibility to different antibiotics was determined in relation to the total *E. coli* population.

### Phylogenetic Groups, Virulence Genotyping, ST131 Detection, and Enterobacterial Intergenic Repetitive Element Sequences (ERIC)-PCR

Phylogenetic groups of *E. coli* isolates were determined by quadruplex PCR and by employing the criteria as described by Clermont et al. (2013). All isolates were screened for the presence of the following five ExPEC genes (Hussain et al., 2014); *papC* (P fimbriae), *sfa* (s fimbriae), *afa* (afimbrial adhesin), *aer* (siderophore related protein), and *cvaC* (protectin). The criteria elaborated by Johnson et al. (2003) were used to classify *E. coli* isolates as ExPEC with some modifications. All *E. coli* isolates were screened for ST131 gene specific PCR as described elsewhere (Hussain et al., 2014). ERIC-PCR based fingerprint analysis was performed as described in our earlier study (Hussain et al., 2014). The ERIC-PCR bands obtained were analyzed using BioNumerics software (Applied Maths, Belgium) (Hussain et al., 2014).

### Whole Genome Sequencing (WGS) and Comparison with Human *E. coli* Pathotypes

Paired end sequencing of the 10 genetically distinct (based on ERIC bands), and randomly selected MDR-ESBL producing poultry *E. coli* (seven cecum and three meat) isolates was carried out using Illumina MiSeq. The accession numbers of 10 poultry *E. coli* genomes including their genomic features are represented in Supplementary Tables S1 and S4. Paired end read data were filtered for high quality reads followed by *de novo* assembly using NGS QC Toolkit (v2.3.3) (Patel and Jain, 2012) and SPAdes Genome Assembler (v3.6.1) (Bankevich et al., 2012), respectively. The *de novo* contigs thus generated were ordered and scaffolded using Contig-Layout-Authenticator (Shaik et al., 2016). Final draft genomes were obtained by merging the scaffolds using a series of N’s and then submitted to the RAST (Overbeek et al., 2014) server for annotation. The genome statistics were gleaned using ARTEMIS (Rutherford et al., 2000). The sequence type (ST) of each of these strains were determined by *in silico* MLST as used/described elsewhere (Ranjan et al., 2015). Mobile genetic elements as reported in the ExPEC strain EC958 (Totsika et al., 2011) were used as reference to compare the 6 *E. coli* genomes using BRIG (Alikhan et al., 2011). A core-genome-based phylogenetic tree of 50 *E. coli* strains (10 in house poultry *E. coli* genomes, 10 healthy broiler chicken *E. coli* genomes from public sources, 10 APEC, 10 ExPEC and 10 enteric pathogens) (Supplementary Tables S1 and S3) was constructed using Harvest (Treangen et al., 2014) and the resulting tree was visualized using iTol^1^. Virulence and Resistance profiles of these 50 strains were generated and hierarchical clustering was performed using the gplot package of R as described/used by us before (Ranjan et al., 2016).

### Statistical Analysis

Statistical analysis for prevalence of antimicrobial resistance, phylogenetic groups, ESBL and virulence genes were carried out using Fisher’s exact two-tailed test. Statistical analyses for aggregate resistance and virulence scores were carried out using the non-parametric Mann–Whitney *U*-test. Both the tests were implemented in the Statistical Package for the Social Sciences (SPSS, version 10.0). *P*-values ≤ 0.05 were considered as statistically significant.

## Results

### Over Half of the Chicken Meat Samples Were Contaminated with *E. coli*

A total of 168 *E. coli* isolates were recovered from 120 poultry samples using both unsupplemented and antibiotic supplemented (ciprofloxacin-plus-cefotaxime) EMB agar, encompassing 105 and 63 isolates, respectively (Supplementary Table S2). Out of the 32 and 13 raw meat samples from broiler and free-range chicken, 29 (91%) and 11 (84%) were contaminated with *E. coli*, respectively. Further, compared to broiler chicken meat (78%), free-range chicken meat demonstrated lower contamination (15%) by *E. coli* when screened on dual antibiotic supplemented plates (ciprofloxacin-plus-cefotaxime) (Supplementary Table S2).

### Resistance to Empirically Used Antibiotics Was Rampant in Poultry *E. coli*

Across the entire dataset of poultry *E. coli* isolates, resistance to tetracycline was most prevalent (84%) followed by ciprofloxacin (70%), co-trimoxazole (45%), and gentamicin (32%) whereas a small fraction of total *E. coli* were found to be resistant to chloramphenicol (8%) and fosfomycin (4%) (**Table 1**). Within each category of chicken samples (broiler and free-range), the isolation sources (ceca and raw meat) did not vary with respect to antimicrobial resistance profiles (*P* > 0.05 for all variables between groups 1 and 3; and groups 2 and 4) (**Table 1**). However, the two categories of chicken *E. coli* isolates exhibited significant difference in prevalence of resistance (MDR and aggregate resistance scores). Strains of *E. coli* samples in the free-range category tended to be resistant to fewer antimicrobial agents (**Table 1**). Overall, the rates of ESBL producers in broiler and free-range chicken were as follows: meat 46%; ceca 40% and meat 15%; ceca 30%, respectively (**Table 1**). The prevalence of ESBL genes differed insignificantly among the four ESBL positive *E. coli* groups (**Table 1**).

**Table 1:** Antimicrobial susceptibility profiles and aggregate resistance scores for 168 *Escherichia coli* isolates together with molecular characteristics of 63 extended-spectrum-β-lactamase (ESBL)-producing *E. coli*.

Specific trait		No. (%) of isolates resistant				^a^*P*-value*	
	Total (*n* = 168)	Broiler chicken meat isolates (group 1; *n* = 54)	Free-range chicken meat isolates (group 2; *n* = 13)	Broiler chicken ceca isolates (group 3; *n* = 55)	Free-range chicken ceca isolates (group 4; *n* = 46)	Group 1 vs. 2	Group 3 vs. 4
**ABST^e^**							
Tetracycline	141 (84)	50 (93)	12 (92)	54 (98)	25 (54)	ns	0.000
Ciprofloxacin	118 (70)	52 (96)	2 (15)	40 (73)	24 (52)	0.000	0.033
Co-trimoxazole	76 (45)	33 (61)	1 (8)	26 (47)	16 (35)	0.001	ns
Gentamicin	54 (32)	23 (43)	1 (8)	21 (38)	9 (20)	0.018	0.041
Chloramphenicol	13 (8)	5 (9)	0	8 (14)	0	ns	0.007
Fosfomycin	6 (4)	3 (6)	0	3 (5)	0	ns	ns
ESBL phenotype^c^	63 (37)	25 (46)	2 (15)	22 (40)	14 (30)	0.013	ns
MDR Phenotype^d^	85 (51)	37 (68)	1 (8)	35 (64)	12 (26)	0.000	0.000

**ESBL positives/genes**	***n* = 63**	***n* = 25**	***n* = 2**	***n* = 22**	***n* = 14**	^a^*P*-value*	

*bla*_CTX-M-15_	50 (79)	21 (84)	2 (100)	17 (77)	10 (71)	ns	ns
*bla*_TEM_	40 (63)	17 (68)	2 (100)	11 (50)	10 (71)	ns	ns
*bla*_SHV_	20 (32)	8 (32)	1 (50)	6 (27)	5 (36)	ns	ns

**Aggregate antimicrobial resistance scores**		**Score median (range)**				^b^*P*-value*	

	3 (0–6)	3 (0–5)	1 (0–4)	3 (0–6)	2 (0–4)	0.000	0.000

*^∗^All comparisons between the groups 1 and 3, and groups 2 and 4 were non-significant (ns) (*P* > 0.05).*

*^a^P-values by Fisher’s exact test, two-tailed.*

*^b^P-values (Mann–Whitney *U*-test) are shown between aggregate resistance scores (sum of resistance genes detected).*

*^c^Extended-spectrum-β-lactamase.*

*^d^Multidrug-resistant.*

*^e^Antibiotic susceptibility tests.*

### Broiler Chicken Was a Potential Source of Pathogenic *E. coli* Variants Than the Free-Range Chicken

The distribution of poultry *E. coli* isolates with respect to the seven phylogenetic groups is shown in **Table 2**. Overall, the majority of poultry *E. coli* isolates were affiliated to group A and B1 (36% each), followed by group D (9%), C (8%), F (7%), E and B2 (2%, each). The prevalence of virulence genes among broiler and free-range chicken *E. coli* isolates was stratified, but the significant prevalence difference (*P* = 0.000) was only observed between the ceca isolates of the above two categories (**Table 2**). Overall, 5% (5/109) of broiler *E. coli* isolates were identified to be extraintestinal pathogenic *E. coli* (ExPEC) based on the detection of three or more ExPEC virulence markers. However, none of the free-range chicken *E. coli* isolates was suspected to be belonging to ExPEC. Similarly, we found one putative ST131 *E. coli* in broiler category, which was later confirmed by WGS and *in silico* MLST, whereas none (0/59) of the free-range *E. coli* isolates belonged to ST131 clone.

**Table 2:** Overall prevalence of phylogenetic group and virulence gene distribution in 168 *Escherichia coli* isolates recovered from both broiler and free-range chicken with aggregate virulence scores.

Specific trait		No. (%) of isolates with trait				*^a^P*-value			
	Total (*n* = 168)	Broiler chicken meat isolates (group1; *n* = 54)	Free-range chicken meat isolates (group2; *n* = 13)	Broiler chicken ceca isolates (group 3; *n* = 55)	Free-range chicken ceca isolates (group 4; *n* = 46)	Group 1 vs. 2	Group 3 vs. 4	Group 1 vs. 3	Group 2 vs. 4
**Phylogenetic group**									
A	61 (36)	19 (35)	7 (54)	10 (18)	25 (54)	ns	0.000	0.045	ns
B1	60 (36)	22 (41)	1 (8)	22 (40)	15 (33)	0.024	ns	ns	ns
B2	3 (2)	1 (2)	0	0	2 (4)	ns	ns	ns	ns
C	13 (8)	3 (6)	0	6 (11)	4 (9)	ns	ns	ns	ns
D	15 (9)	5 (9)	2 (15)	8 (15)	0	ns	0.007	ns	0.007
E	3 (2)	1 (2)	1 (8)	1 (2)	0	ns	ns	ns	ns
F	11 (7)	3 (6)	2 (15)	6 (11)	0	ns	0.021	ns	0.007
**Virulence genes**									
*aer*	51 (30)	26 (48)	0	25 (45)	0	0.001	0.000	ns	–
*afa*	17 (10)	8 (15)	1 (8)	6 (11)	2 (4)	ns	ns	ns	ns
*papC*	6 (4)	4 (7)	0	2 (4)	0	ns	ns	ns	–
*cvaC*	29 (17)	6 (11)	5 (38)	13 (24)	5 (11)	0.017	ns	ns	0.019
*sfa*		0	0	0	0				
ExPEC^c^	5 (3)	4 (7)	0	1 (2)	0	ns	ns	ns	–

**Aggregate virulence scores**		**Score median (range)**				***^b^P*-value**			

		1 (0–3)	0 (0–2)	1 (0–3)	0 (0–2)	ns	0.000	ns	0.010

*^a^*P*-values by Fischer’s exact test, two-tailed.*

*^b^P-values (Mann–Whitney *U*-test) are shown between aggregate virulence scores (sum of virulence markers detected).*

*^c^ExPEC status is defined if ≥ 3 of the virulence genes are detected in an organism.*

*ns, non-significant (*P*-values > 0.05).*

### Multiple Chicken Samples from Same Shops Harbored Identical *E. coli* Clones

**FIGURE 1:**
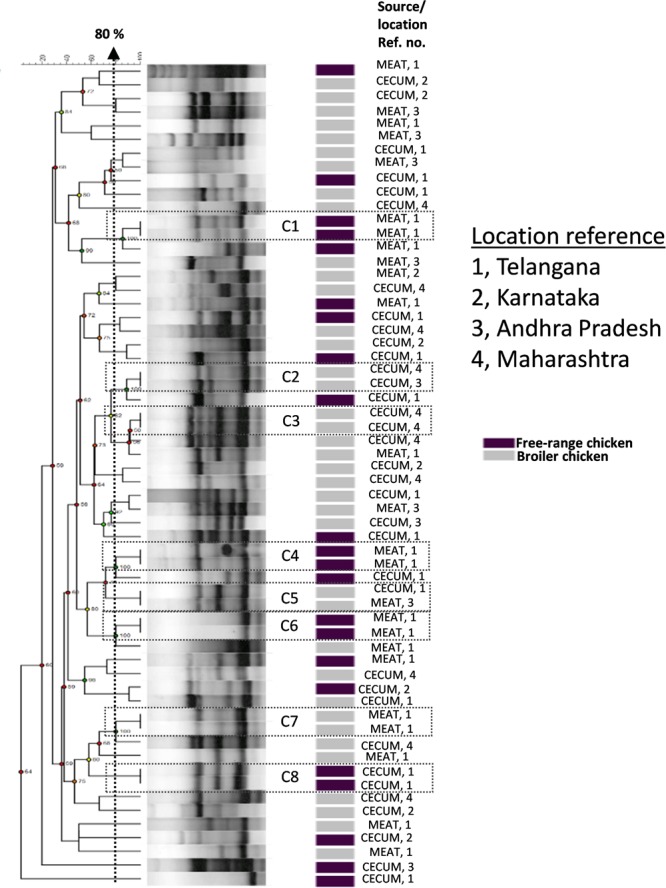
Dendrogram representing genetic relationships of 38 broiler and 22 free-range chicken *Escherichia coli* isolates based on ERIC-PCR fingerprints. The clustering was performed by UPGMA algorithm based on Dice similarity coefficients. The genotypic patterns generated by ERIC-PCR were analyzed at 80% cutoff. Among the eight identical clades (C1 to C8), six clades corresponded consistently to geographic and isolation source.

We investigated the genetic relationships among the *E. coli* isolates obtained from broiler and free-range chicken samples (**Figure 1**). A total of 60 isolates that were multidrug resistant and originating from different geographical locations were analyzed that represented 38 broiler (22 ceca and 16 raw meat) and 22 free-range chicken isolates (12 ceca and 10 raw meat). Out of 60 isolates, 52 distinct ERIC profiles were obtained. Overall, the broiler and free-range chicken *E. coli* isolates demonstrated promiscuous genetic fingerprints as the two categories did not form distinct clusters. A total of 39 out of 60 isolates demonstrated a close genetic relatedness (similarity coefficient of ≤80%) which were represented in the form of several small sub-clusters. Six out of eight identical clades corresponded consistently with the geographic origin, isolation source and ESBL status but not the sample origin; this possibly hints at cross contamination during rearing, slaughtering and/or processing.

### Poultry *E. coli* Isolates Shared Remarkable Similarities with Human and Avian *E. coli* Pathotypes

**FIGURE 2:**
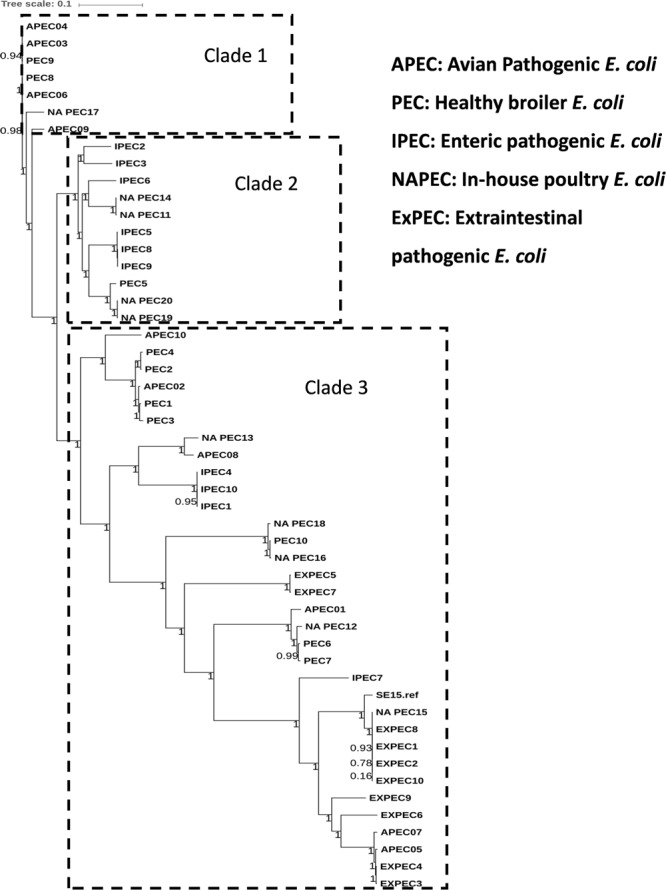
Phylogenetic tree of 50 *E. coli* strains: the core genome based consensus Maximum Likelihood phylogenetic tree of 10 in-house poultry *E. coli* isolates together with 40 other publicly available human and animal disease associated *E. coli* isolates generated using Harvest. The output of Harvest was visualized using iTOL tool (http://itol.embl.de/). Despite genetic diversity, the phylogenetic tree on the whole was able to diffusely group isolates belonging to different pathotypes.

The general features of 10 in-house whole genome sequenced poultry *E. coli* isolates were shown in Supplementary Tables S1 and S4. We generated a phylogenetic tree (**Figure 2**) of 10 in-house isolates to study their relationship with 40 other publicly available genomes comprising of 20 human disease-associated *E. coli* (10 ExPEC and 10 *enteric* pathogens), 10 Avian pathogenic *E. coli* (APEC) and 10 *E. coli* genomes from healthy broiler chickens (Supplementary Table S3). Despite genetic diversity, the phylogenetic tree was able to largely cluster isolates of different pathotypes. The clade 1 was dominated with avian strains and clade 2 mainly comprised genomes of enteric strains together with poultry *E. coli*. The clade 3 comprised strains from all five pathotypes in which the in-house poultry *E. coli* was found to be co-clustered with APEC, poultry and ExPEC strains but not with enteric strains. Out of 10 poultry genomes analyzed, one was represented in clade 1, four genomes in clade 2 and five genomes in clade 3. Overall, we observed that the core genome content of poultry *E. coli* genomes failed to demonstrate unambiguous distinction with other human *E. coli* pathotypes.

**FIGURE 3:**
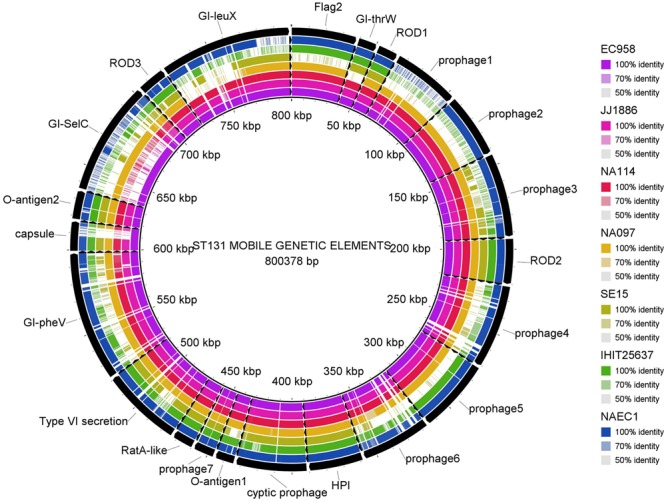
Distribution of EC958 genomic islands (GIs) in 6 ST131 *E. coli* genomes: results show that the 22 GIs of EC958 are well-conserved among three human and one in-house ST131 poultry *E. coli* genome (NAEC1) but partially present in the commensal ST131 (SE15) and the poultry *E. coli* genome from Germany (IHIT25637). Detail description of the genomes analyzed are as follows- *JJ1886*; CP006784.1 (Human ExPEC, United States), *NA114*; GCA_000214765.3 (Human ExPEC, India), *NA097*; GCA_001029415.1 (Human ExPEC, India), *SE15*; GCA_000010485.1 (Human commensal, Japan), *IHIT25637*; GCA_001676995.1 (Avian ExPEC, Germany).

Multiple genome comparison was carried out using BRIG tool for one in-house poultry *E. coli* isolate (NAEC1 or NAPEC_15) belonging to the ST131 lineage with five other ST131 *E. coli* genomes to determine the status of 22 mobile genetic elements as reported in EC958 (**Figure 3**). Out of six genomes, three strains (JJ1886, NA114, and NA097) including the NAEC1 shared significant genetic similarities with respect to composition of mobile genetic elements. However, the other avian genome IHT25637 and the commensal ST131 genome SE15 contained only few complete mobile elements. The presence of mobile genomic islands in the poultry *E. coli* resembling to that of human *E. coli* pathotypes reinforces the understanding that these isolates could possibly cross infect humans and poultry.

**FIGURE 4:**
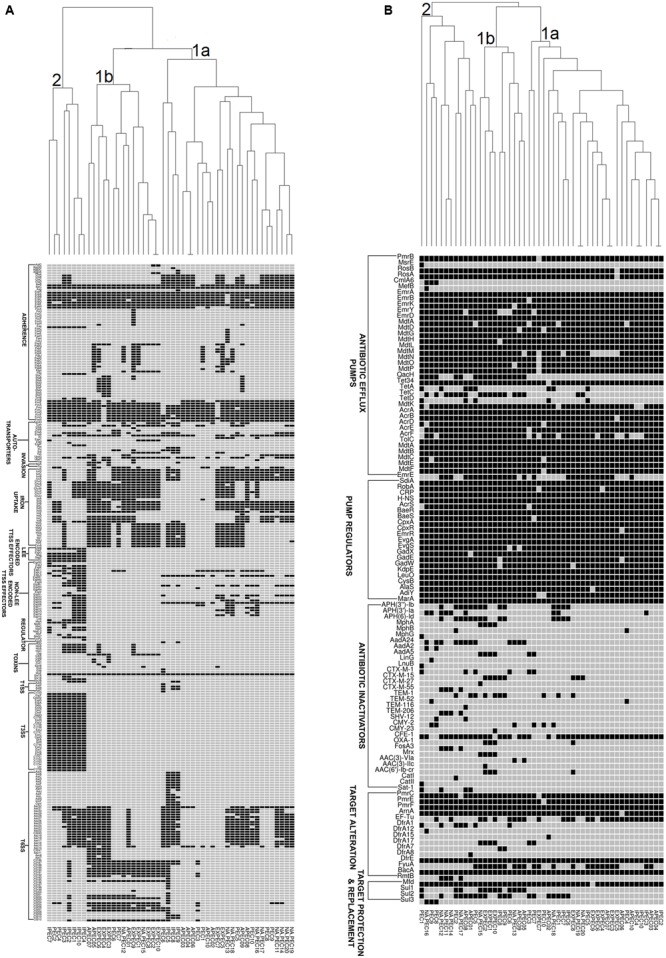
Gene cluster results for 50 *E. coli* isolates: the presence (Black Square) and the absence (Gray Square) of virulence genes **(A)** and resistance genes **(B)** are represented in the image. Gene names are listed on the left. *E. coli* pathotypes are listed below the image (APEC: avian pathogenic *E. coli*, ExPEC: extraintestinal pathogenic *E. coli*, IPEC: intestinal pathogenic *E. coli*, PEC: healthy poultry *E. coli*, NA_PEC: in-house poultry *E. coli*). Results of resistance clustering indicated that only 50% of our poultry *E. coli* shared resistance genes with other *E. coli* pathotypes (distributed in mixed clusters 1a and 1b) and the rest formed a separate group (cluster 2, **B**). However, the virulence based cluster diagram showed that the in-house poultry (NAPEC) shared more virulence similarity with ExPEC and APEC genomes compared to intestinal pathogenic *E. coli* (IPEC), as cluster 2 of **(A)** was dominated by enteric *E. coli* genomes without any poultry *E. coli* genome.

In order to understand the genetic relationships of virulence and resistance of poultry *E. coli* in juxtaposition with the genetic landscapes of different human *E. coli* pathotypes, we compared virulence and resistance profiles of 10 sequenced poultry *E. coli* genotypes with 40 publicly *E. coli* genomes (Supplementary Table S3). Hierarchical clustering of resistance profiles (**Figure 4B**) demonstrated that the two sister clusters 1a and 1b represented isolates from all pathotypes including the five in-house poultry genomes. However, cluster 2 was clearly defined or dominated by poultry *E. coli* isolates that also contained the remaining five in-house poultry genomes. The cluster obtained by the virulence gene profiles (**Figure 4A**) grouped strains into two categories; one composed of mixed pathotypes (sister clusters 1a and 1b) and the other cluster 2 was dominated with enteric isolates (8/10 enteric genomes). The 10 in-house poultry *E. coli* isolates were represented in the mixed clusters (1a and 1b) indicating closer genetic identity with some pathotypes (ExPEC, APEC and poultry *E. coli*), than with others (IPEC or enteric pathotypes). Overall, these observations hint at the zoonotic potential of poultry *E. coli.*

## Discussion

In this molecular-epidemiological study, we assessed *E. coli* isolates obtained from retail poultry (meat/carcass) from a wide geographical region extending across three south Indian and one west-central Indian states (four states) by using both conventional typing and WGS methods. Our findings provide evidence that the raw retail poultry meat could indeed be contaminated with antimicrobial-resistant and potentially pathogenic *E. coli.* This is particularly alarming for countries such as India given high disease burden, emergence of resistance traits, and the confluence of prevailing socio-economic, demographic and environmental factors (Jadhav et al., 2011; Dikid et al., 2013).

Pathogenic *E. coli* (ExPEC and diarrheagenic) are the leading cause of infections in both humans and poultry (Ewers et al., 2007). Increased antimicrobial usage in the poultry industry due to the growing demand might have contributed significantly to the emergence and dissemination of multiresistant and pathogenic *E. coli* variants, which could be a serious public health threat (Laxminarayan and Chaudhury, 2016). Hence, constant surveillance and knowledge of their transmission and epidemiology with respect to their genetic backgrounds, antimicrobial resistance patterns and specific virulence attributes is pertinent (Mellata, 2013; Laxminarayan and Chaudhury, 2016). Our findings therefore have potential implications for public health policies entailing antibiotic usage regulation.

Poultry industries use antibiotics both for therapeutic purposes and for growth promotion (Singer and Hofacre, 2006). Recent studies in different parts of India have reported antimicrobial residues in food animal products such as chicken meat suggesting large-scale unregulated use of antibiotics by the poultry industry (Laxminarayan and Chaudhury, 2016; Brower et al., 2017). This is consistent with our observations as we also found a marked predominance of antibiotic resistance among *E. coli* isolates obtained from conventionally raised (broiler) chicken. In contrast, free-range chicken meat were comparatively less contaminated with multidrug resistant *E. coli*; this observation echoes previously reported studies (Koga et al., 2015; Rugumisa et al., 2016). The above observation complements the cross contamination hypothesis of poultry carcasses with the host’s fecal flora during slaughter and processing also given that we found comparable antimicrobial resistance prevalences in *E. coli* isolates entailing ceca and raw meat samples of broiler chicken. Such observations were also evidenced by others (Rasschaert et al., 2008; Firildak et al., 2015). The reason for higher prevalence of antimicrobial resistance in *E. coli* isolates of broiler chicken could be due to the high antibiotic selection pressure.

Similar to other studies (Klimienė et al., 2017) the predominant ESBL genotype detected in chicken *E. coli* isolates was *bla*_CTX-M-15_ gene which is also the most frequently reported genotype in human health care settings (Chen et al., 2014). The pan-genome based resistance gene profiling revealed that 50% of poultry *E. coli* shared resistance genes with human *E. coli* pathotypes (**Figure 4B**). The formation of a separate cluster of the remaining 50% of the poultry genomes indicates difference in resistance determinants with that of human *E. coli* resistomes which could be reflective of differences in antimicrobial usage practices in human medicine and livestock rearing.

Although only a fraction of total poultry isolates did belong to two well-established pathogenic phylogroups B2 and D (2%, 9%, respectively), a majority of them (36% each) belonged to phylogroups A and B1 (**Table 2**), as also reported by others (Jakobsen et al., 2010; Klimienė et al., 2017). Nonetheless, these two phylogroups have also been previously described to harbor isolates with a high pathogenic potential for birds and humans (Rodriguez-Siek et al., 2005; Ewers et al., 2007; Kobayashi et al., 2011). Virulence gene (Franz et al., 2015) screening demonstrated that the broiler *E. coli* isolates differed from free-range chicken *E. coli* isolates by their higher prevalence of all the virulence genes. Furthermore, five broiler *E. coli* isolates were identified to be ExPEC with none from free-range chicken *E. coli* (**Table 2**). The overall low prevalence of poultry *E. coli* isolates from ExPEC category was expected because all samples were collected from healthy birds. This corroborates also with the low prevalence of phylogenetic group B2.

The phylogenetic tree, based on core genomes demonstrated that, the poultry *E. coli* isolates are not a homogeneous group, because some of the poultry *E. coli* isolates revealed similar genetic backgrounds with human ExPEC and enteric isolates, thus pointing to the role of chickens as reservoir of potentially pathogenic *E. coli*. Only a few studies have investigated the presence of *E. coli* ST131 in food animals; *E. coli* ST131 producing CTX-M-15 also appears to be very rare in foodstuffs of animal origin (Nicolas-Chanoine et al., 2014). This study confirmed the presence of two globally emerging pathogenic lineages of *E. coli*; ST131 (*H*30-Rx subclone) and ST117. The other commonly identified sequence types among the 10 poultry *E. coli* genomes were; ST115, ST155, and ST1640 which are reported to be mostly associated with ESBL phenotypes (Borjesson et al., 2013). Moreover, the identified ST131 *E. coli* contained more pathogenicity islands than the other poultry genomes from Europe and the commensal ST131 genome-SE15 (**Figure 3**). The presence of such a strain in poultry meat belonging to the epidemiologically successful clonal lineage ST131 (*H*30-Rx subclone) warrants attention.

Corroborating with phylogeny results, virulence gene profiling revealed that the poultry *E. coli* are not a homogenous group and showed mixed clustering. However, it provided better resolution between ExPEC and enteric pathotypes (**Figure 4A**). Nonetheless, a majority of poultry *E. coli* isolates and human ExPEC were clustered together with respect to the virulence gene content suggesting that they share remarkable similarities with human ExPEC pathotypes (**Figure 4A**). The ERIC fingerprinting of broiler and free-range chicken *E. coli* isolates demonstrated that the poultry *E. coli* are diverse. The identical clones that we observed mostly corresponded to the geographic (abattoir) and host (broiler and free-range) origins (**Figure 1**).

This geographically wide report on molecular and genomic characterization of *E. coli* from broiler and free-range chickens is the first from India. Herein, we found that the raw retail poultry meat was frequently contaminated with antimicrobial-resistant *E. coli* and/or potentially pathogenic *E. coli* variants. However, free-range chickens represented a low risk of contamination with pathogenic and or resistant *E. coli*; this is particularly important as India is an agricultural country with around 70% of the population living in rural areas and many of the rural households engage in backyard poultry raising (Singh, 2014). The comparative genomic analysis suggests that the poultry *E. coli* isolates share closer genetic identity to human *E. coli* as regards to core genome phylogeny, antimicrobial genes and virulence gene content and thus represent potential zoonotic risks. The possibility that multi-drug resistant and /or pathogenic *E. coli* could be potentially transmitted from food sources such as chicken meat raises serious public health concerns regarding food preparations where chicken meat may not be properly cooked or could only be pickled raw.

## Author Contributions

AH, DC, and NA designed and conducted the study. AR, NN, TS, LW, MI, MM, and IQ helped in analysis of data and preparation of the manuscript. SS, ST, and RB helped in analyzing genome sequence data.

## Conflict of Interest Statement

The authors declare that the research was conducted in the absence of any commercial or financial relationships that could be construed as a potential conflict of interest.

## Footnotes

1 http://itol.embl.de/
