# Supplementary material for: Risk of Transmission of Antimicrobial Resistant Escherichia coli from Commercial Broiler and Free-Range Retail Chicken in India
Source: Front Microbiol. 2017 Nov 13;8:2120. doi: 10.3389/fmicb.2017.02120 (PMC5694193; doi:10.3389/fmicb.2017.02120)
Supplement: Supplementary file 1 [file Table_1.PDF]

Table S1: Genome features and assembly statistics of 10 whole genome sequenced poultry *E. coli* isolates

| Genome | Accession Number | Isolation source | No. of Raw reads | Genome coverage (X) | Number of contigs >=500bp | N50 value | Sequence Type (ST) | G+C content (%) | Genome size (bp) | Coding % | No. of CDS | No. of rRNAs |
|---|---|---|---|---|---|---|---|---|---|---|---|---|
| **NA1001** | MKWV00000000 | Broiler cecum | 457192 | 21.9 | 232 | 79906 | UNK | 50.41 | 5230886 | 85.8 | 5198 | 10 |
| **NA1002** | MKWW00000000 | Broiler cecum | 1330724 | 69.7 | 187 | 123923 | ST117 | 50.72 | 5256107 | 86.3 | 5227 | 16 |
| **NA1003** | MKWX00000000 | Broiler cecum | 1430266 | 73.8 | 257 | 86543 | ST1640 | 50.84 | 5461579 | 86.1 | 5444 | 13 |
| **NA1004** | MKWY00000000 | Broiler cecum | 1580522 | 80.3 | 167 | 165460 | UNK | 50.48 | 5291577 | 86.3 | 5229 | 18 |
| **NAEC1** | MLAZ00000000 | Broiler meat | 2197830 | 60.8 | 138 | 163214 | ST131 | 50.73 | 5152191 | 87.4 | 5037 | 13 |
| **NAEC2** | MLBA00000000 | Broiler cecum | 1779900 | 61.7 | 222 | 88992 | ST115 | 50.36 | 5507065 | 85.9 | 5398 | 7 |
| **NAEC3** | MLBB00000000 | Broiler meat | 1446944 | 52.8 | 181 | 111651 | UNK | 50.47 | 5144964 | 85.9 | 5084 | 10 |
| **NAEC4** | MLBC00000000 | Broiler meat | 1806644 | 62.1 | 276 | 94921 | ST-115 | 50.37 | 5786021 | 85.4 | 5766 | 9 |
| **NAEC5** | MLBD00000000 | Broiler cecum | 1189470 | 41.6 | 193 | 124307 | ST-155 | 50.45 | 5195760 | 85.8 | 5025 | 14 |
| **NAEC6** | MKWZ00000000 | Broiler cecum | 1377018 | 46.5 | 119 | 123677 | ST-155 | 50.48 | 5095482 | 87 | 4948 | 12 |

UNK; Unknown sequence type

Table S2: Details of the poultry *E. coli* obtained with respect to isolation source and chicken category

| | Broiler ceca n=39 | Broiler meat n=32 | Free-range ceca n=36 | Free-range meat n=13 |
|---|---|---|---|---|
| *E. coli* on unsupplemented media | 33 (85%) | 29 (91%) | 32 (89%) | 11 (84%) |
| *E. coli* on supplemented media | 22 (56%) | 25 (78%) | 14 (39%) | 2 (15%) |

Table S3: List of *E. coli* genomes used in this study

| S. No. | Accession | Strain name | In-house ID |
|---|---|---|---|
| 1 | AOGL00000000 | SEPT362 | APEC01 |
| 2 | AOGN00000000 | S17 | APEC02 |
| 3 | CP007442 | ACN001 | APEC03 |
| 4 | CP004009 | APEC_O78 | APEC04 |
| 5 | CP000468 | APEC_O1 | APEC05 |
| 6 | HE964769 | IMT2125 | APEC06 |
| 7 | CP005930 | APEC_IMT5155 | APEC07 |
| 8 | LHAT00000000 | INSLA289 | APEC08 |
| 9 | CP010315 | 789 | APEC09 |
| 10 | CP013048 | RS76 | APEC10 |
| 11 | ASHA00000000 | NCCP15739 | IPEC1 |
| 12 | AP010958 | 12009 | IPEC2 |
| 13 | AP010953 | 11368 | IPEC3 |
| 14 | AE005174 | EDL933 | IPEC4 |
| 15 | CU928145 | 55989 | IPEC5 |
| 16 | CP000800 | E24377A | IPEC6 |
| 17 | FM180568 | E2348_69 | IPEC7 |
| 18 | NZ_AFWP00000000 | GOS2 | IPEC8 |
| 19 | NZ_AFWO00000000 | GOS1 | IPEC9 |
| 20 | NC_002695 | Sakai_RIMD_0509952 | IPEC10 |
| 21 | MIPU00000000 | NA114 | EXPEC1 |
| 22 | JSXJ00000000 | NA097 | EXPEC2 |
| 23 | CP001969 | IHE3034 | EXPEC3 |
| 24 | NC_007946 | UTI89 | EXPEC4 |
| 25 | NC_011750 | IAI39 | EXPEC5 |
| 26 | JSVN00000000 | UPEC_008 | EXPEC6 |
| 27 | CP003034 | CE10 | EXPEC7 |

| | | | |
|---|---|---|---|
| 28 | CP006784 | JJ1886 | EXPEC8 |
| 29 | AE014075 | CFT073 | EXPEC9 |
| 30 | HG941718 | EC958 | EXPEC10 |
| 31 | AYQO00000000 | 38.27 | PEC1 |
| 32 | AYQP00000000 | 38.34 | PEC2 |
| 33 | AYQQ00000000 | 53A | PEC3 |
| 34 | AYQR00000000 | 85B | PEC4 |
| 35 | AYQL00000000 | 38.16 | PEC5 |
| 36 | AYQH00000000 | 38.52 | PEC6 |
| 37 | AYRA00000000 | 53C | PEC7 |
| 38 | AYQX00000000 | 1A | PEC8 |
| 39 | AYQZ00000000 | 27A | PEC9 |
| 40 | AYRB00000000 | 87A | PEC10 |
| 41 | MKWV00000000 | NA1001 | NA_PEC11 |
| 42 | MKWW00000000 | NA1002 | NA_PEC12 |
| 43 | MKWX00000000 | NA1003 | NA_PEC13 |
| 44 | MKWY00000000 | NA1004 | NA_PEC14 |
| 45 | MLAZ00000000 | NAEC1 | NA_PEC15 |
| 46 | MLBA00000000 | NAEC2 | NA_PEC16 |
| 47 | MLBB00000000 | NAEC3 | NA_PEC17 |
| 48 | MLBC00000000 | NAEC4 | NA_PEC18 |
| 49 | MLBD00000000 | NAEC5 | NA_PEC19 |
| 50 | MKWZ00000000 | NAEC6 | NA_PEC20 |

Table S4: Results of antimicrobial susceptibility, virulence gene profiling and phylogenetic group determination of 10 whole genome sequenced poultry *E. coli*

| **Genome** | **ABST** | | | | | | **Phylogenetic groups** | **Virulence genes** | | | | |
|---|---|---|---|---|---|---|---|---|---|---|---|---|
| | **COT** | **TET** | **CIP** | **C** | **G** | **F** | | ***aer*** | ***afa*** | ***cvaC*** | ***papC*** | ***sfa*** |
| **NA1001** | S | R | R | S | R | S | B1 | + | - | - | - | - |
| **NA1002** | R | R | R | S | R | S | F | + | - | + | + | - |
| **NA1003** | R | R | R | S | S | S | D | + | - | + | - | - |
| **NA1004** | S | R | R | S | R | S | B1 | + | - | - | - | - |
| **NAEC1** | R | R | R | S | R | S | B2 | + | - | + | + | - |
| **NAEC2** | R | R | R | R | R | S | D | + | - | - | - | - |
| **NAEC3** | S | R | R | S | S | S | A | + | - | + | - | - |
| **NAEC4** | S | R | R | S | S | S | B1 | + | - | - | - | - |
| **NAEC5** | S | R | R | S | S | S | B1 | + | - | - | - | - |
| **NAEC6** | R | R | R | S | R | R | A | - | - | - | - | - |

ABST: antimicrobial susceptibility

CoT; Cotrimoxazole, TET; Tetracycline, CIP; Ciprofloxacin, C; Chloramphenicol, G; Gentamicin, F; Fosfomycin

*papC* (P fimbriae), *sfa* (s fimbriae), *afa* (afimbrial adhesin), *aer* (siderophore related protein) and *cvaC* (protectin)
